# Resonance Raman Spectroscopic Study of the Unusual [4Fe‐4S]^2+^ Cluster of IspH, the Last Enzyme of the Methylerythritol Phosphate Pathway for Terpenoid Biosynthesis

**DOI:** 10.1002/cbic.202500428

**Published:** 2025-08-26

**Authors:** Hannah Jobelius, Philippe Chaignon, Gabriella I. Bianchino, Joanna Wandzig, Petra Hellwig, Myriam Seemann, Frederic Melin

**Affiliations:** ^1^ Equipe Chimie Biologique et Applications Thérapeutiques Institut de Chimie de Strasbourg, UMR 7177 CNRS Strasbourg 67000 France; ^2^ Laboratoire de Bioélectrochimie et Spectroscopie UMR 7140 Chimie de la Matière Complexe Université de Strasbourg CNRS Strasbourg 67000 France; ^3^ Institut Universitaire de France (IUF) Strasbourg 67000 France

**Keywords:** 4Fe‐4S clusters, IspH, methylerythritol phosphate pathway, Raman spectroscopy, terpenoids

## Abstract

IspH is the last enzyme of the methylerythritol phosphate pathway. It catalyzes the reductive dehydroxylation of (*E*)‐4‐hydroxy‐3‐methyl‐but‐2‐en‐1‐yl diphosphate into isopentenyl diphosphate (IPP) and dimethylallyl diphosphate (DMAPP), which are precursors for the biosynthesis of terpenoids, essential molecules for the survival of all living organisms. This pathway is absent in humans, making it a promising target for drug discovery. *Escherichia* *coli* IspH harbors an unusual [4Fe‐4S]^2+^ cluster linked to three conserved cysteines with a unique iron site proposed to be coordinated to three water molecules. Here, the first resonance Raman spectroscopic study of the cluster of IspH in the 2+ oxidation state is reported. Using isotopic labeling with ^2^H_2_O and H_2_^18^O, the bands of the cluster that are sensitive to water coordination or hydrogen bonding are identified. The change of geometry of the cluster upon binding of the substrate, an alkyne diphosphate inhibitor, and the two enzyme products is also analyzed. Distinct binding modes to the cluster may indeed be at the origin of the different distribution of IPP and DMAPP observed during catalysis.

## Graphical abstract

Raman spectroscopy confirms that the apical Fe atom of the [4Fe‐4S]^2+^ cluster of IspH is linked to H_2_O molecules and that the (*E*)‐4‐hydroxy‐3‐methyl‐but‐2‐en‐1‐yl diphosphate substrate and the propargyl diphosphate inhibitor lead to a change of coordination geometry. The loss of octahedral coordination of the apical Fe atom is also very likely when the dimethylallyl diphosphate product is added, while no change is observed with isopentenyl diphosphate.

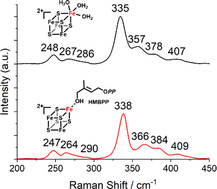

## 1 Introduction

Antimicrobial resistance (AMR) has become one of the major threats^[^ ^1^ ^]^ to global public health. According to a comprehensive analysis that monitored 23 bacterial pathogens and 88 pathogen–drug combinations in 204 countries, bacterial AMR contributed to 4.95 million deaths and was held directly responsible for 1.27 million deaths in 2019.^[^ ^2^ ^]^ It is estimated that the number of deaths attributable to AMR could increase to 10 million by 2050 if AMR is not addressed.^[^ ^3^ ^]^ In addition to being a threat to public health, AMR can have a huge impact on the economy by inducing productivity losses, increased healthcare costs, and can lead to a rise in poverty.

There is thus an urgent need to discover new cellular targets which will hopefully lead to new antimicrobial drugs with novel mechanisms of action.^[^ ^4^ ^]^ The methylerythritol phosphate (MEP) pathway is of great interest for the research of novel antimicrobial agents.^[^ ^5^, ^6^, ^7^ ^–^ ^8^ ^]^ It is found in chloroplasts, pathogenic bacteria, and parasites, such as *Plasmodium falciparum*. The last enzyme of this pathway, which is called LytB or IspH, catalyzes the reductive dehydroxylation of (E)‐4‐hydroxy‐3‐methyl‐but‐2‐en‐1‐yl diphosphate (HMBPP) into two products: isopentenyl diphosphate (IPP) and dimethylallyl diphosphate (DMAPP).^[^ ^9^ ^]^ These are the universal precursors for the biosynthesis of terpenoids, which are essential molecules for the survival of all living organisms. In humans, IPP is produced by the so‐called mevalonate pathway and converted to its isomer DMAPP by type I isopentenyl diphosphate isomerase. The production of IPP and DMAPP in humans is thus independent of IspH, making this enzyme a suitable target for the development of antibacterial and antimalarial agents. To the best of our knowledge, however, no IspH inhibitor was reported, that showed antibacterial activity. These inhibitors are typically substrate analogs containing a diphosphate moiety,^[^ ^9^ ^]^ and thus, have a low membrane permeability. In addition, they can be hydrolyzed by phosphatases before acting as antibacterial agents.

IspH harbors an oxygen‐sensitive [4Fe‐4S]^2+^ cluster that is crucial for its catalytic function.^[^ ^10^ ^,^ ^11^ ^]^ Unlike the prototypical [4Fe‐4S]^2+^ clusters that possess a cubane‐like Td symmetry with four Fe atoms bound to three bridging S^2−^ ions and to one cysteine residue each,^[^ ^12^ ^,^ ^13^ ^]^ the [4Fe‐4S]^2+^ cluster of IspH deviates from this geometry. In IspH, one Fe atom, the so‐called apical Fe, is not coordinated by a cysteine ligand and it exhibits an octaedral geometry leading to a [4Fe‐4S]^2+^ cluster with a C3v symmetry. According to Mössbauer spectroscopy^[^ ^14^ ^,^ ^15^ ^]^ and nuclear‐resonance vibrational spectroscopy (NRVS) combined with density functional theory studies,^[^ ^16^ ^]^ the apical Fe in IspH from *Escherichia coli* is coordinated to three bridging S^2−^ ligands from the cluster, and the remaining ligands are suggested to be three water molecules.

**Figure 1:**
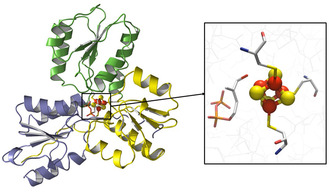
X‐ray crystal structure of *E. coli* IspH in complex with HMBPP (PDB: 3KE8). The structure shows the cloverleaf‐like motif of the three domains depicted in different colors. The [4Fe‐4S]^2+^ cluster is observed in the central cavity of the enzyme.

The first X‐ray crystal structure of IspH from *Aquifex aeolicus,* published by Ermler et al. in 2008, revealed a cloverleaf‐like structure with three *α*/*β* domains.^[^ ^17^ ^]^ Although the crystals were grown under anaerobic conditions, the central [4Fe‐4S]^2+^ cluster was lacking one Fe atom. Groll et al. then published the crystal structure of *E. coli* IspH^[^ ^18^ ^]^ that presented again the cloverleaf‐like structure with a [3Fe‐4S] cluster in the central cavity. **Figure** **1** shows the X‐ray crystal structure of *E. coli* IspH in complex with HMBPP that was reported by the same group and exhibited for the first time the complete [4Fe‐4S] cluster.^[^ ^19^ ^]^ The apical Fe atom adopts a tetrahedral coordination geometry, and HMBPP a hairpin‐like conformation. Several structures of the enzyme with the [4Fe‐4S] cluster bound to different types of inhibitors were also published.^[^ ^20^ ^,^ ^21^ ^]^

**Scheme 1:**
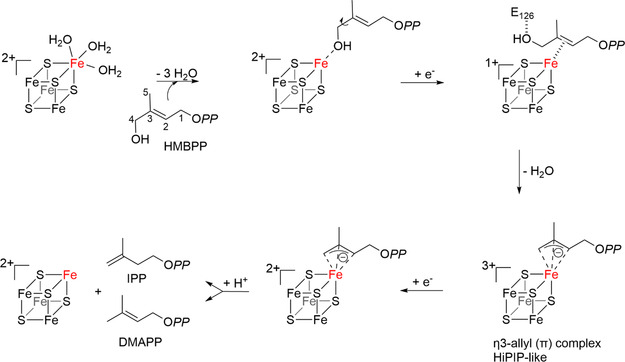
Currently proposed mechanism for the IspH‐catalyzed reaction. O*PP* stands for pyrophosphate group.

A better understanding of the mechanism of action of IspH might lead to new strategies to combat AMR. Studies on the mechanism of this metalloenzyme have thus been conducted for over 20 years, and several proposals were discussed.^[^ ^11^ ^,^ ^22^ ^,^ ^23^ ^]^ Upon substrate binding, the hydroxyl group of HMBPP binds to the unique apical iron atom (see **Scheme** **1**). After reduction of the cluster, it is now generally accepted that the hydroxymethyl group is rotated and a *π*‐complex is formed.^[^ ^24^, ^25^, ^26^ ^–^ ^27^ ^]^ The hydroxyl group is then eliminated as water after the transfer of a proton from the Glu126 residue, and an internal two‐electron transfer occurs from the iron–sulfur cluster to the carbon chain, leading to a *η* ^3^‐allyl complex.^[^ ^11^ ^,^ ^22^ ^]^ The reduction of this latter followed by a protonation either at the C‐2 or at the C‐4 atom leads to the two products IPP and DMAPP.

The last step of the mechanism, however, is not yet fully understood, as the thermodynamically favored product DMAPP is produced in lower quantities than its configurational isomer IPP. It was shown that for *E. coli* IspH, the ratio IPP:DMAPP was about 6:1.^[^ ^28^ ^]^ Also, the redox and ligation states of several cluster‐based species detected after incubation with reductant, addition of a ligand, or during catalysis have not yet been established with certainty.^[^ ^23^ ^]^ We report here the first resonance Raman (RR) spectroscopic study of the [4Fe‐4S]^2+^ cluster of IspH. We focus on the stretching Fe—S modes that are highly sensitive to small changes in cluster geometry and environment^[^ ^29^, ^30^ ^–^ ^31^ ^]^ but are not strongly visible in the NRVS data.^[^ ^16^ ^]^ RR also allows to monitor ligand binding dynamically, which is hard with NRVS. Using isotopic labeling by exchanging ^1^H_2_O/^2^H_2_O and H_2_^16^O/H_2_^18^O, we identify the spectroscopic signature of the Fe–S cluster that is sensitive to water coordination to confirm that the apical Fe atom of the cluster is bound to water molecules. We also analyze the change of geometry of the cluster upon binding of the substrate or each of the two enzyme products, IPP and DMAPP, or an alkyne diphosphate that is a potent IspH inhibitor.

## 2 Results and Discussion

### 2.1 Substrate‐Free IspH

The RR spectrum of *E. coli* IspH was measured both under aerobic and anaerobic conditions in an air‐tight plastic bag filled with argon, but no difference was noticed between the two spectra if the measurement time did not exceed 45 min. The sensitivity of the protein toward O_2_ was thus not an obstacle for the Raman measurements described here.

**Figure 2:**
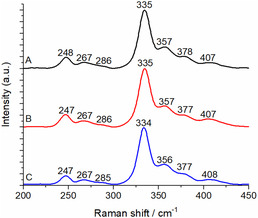
A) RR spectra of ≈0.3 mM IspH, B) IspH in ^2^H_2_O, and C) IspH in H_2_^18^O measured with 457 nm laser excitation and 6 mW laser power at room temperature.

The enzyme shows a broad band at 420 nm in UV/visible spectroscopy, which is ascribed to an S‐to‐Fe charge transfer transition. The Raman experiments were thus performed at an excitation wavelength of 457 nm in order to be in preresonance conditions to increase the sensitivity of the technique. The obtained spectrum (see **Figure** **2A** and the list of peaks in **Table** **1**) is typical for [4Fe–4S]^2+^ clusters with either three or four cysteine ligands.^[^ ^29^, ^32^ ^–^ ^34^ ^]^ It shows a main band at 335 cm^−1^, and lower intensity bands at 248, 267, 357, 378, and 407 cm^−1^ that can all be attributed to Fe–S vibrational modes. According to the literature, these modes can be subdivided into two groups, namely terminal (i.e., Fe–S(Cys)), and bridging Fe–S vibrational modes. No Fe—O stretching vibration was observed here in the 400–600 cm^−1^ range, probably because these vibrations are not enhanced at 457 nm excitation or are overlapped by other contributions.

**Table 1:** Raman shifts (cm^−1^) and tentative assignment of the vibrational modes of IspH observed in the spectra obtained at 457 nm laser excitation.

IspH	IspH/^2^H_2_O	IspH/H_2_^18^O	Aca)	Fd_ox_ a)	IspGa)
Mainly terminal Fe‐S modes					
407	407	408	372	396	387
–	–	–	360	363	–
357	357	356	352	–	352
Mainly bridging Fe—S modes					
378	378	377	392	374	367
335	335	334	339	342	336
–	–	–	299	295	–
286	286	285	287	282	292
–	–	–	275	–	279
267	267	267	269	269	265
248	248	247	255	253	251

a) Comparison with the bands previously reported for [4Fe‐4S]^2+^ clusters of C3v symmetry: aconitase (Ac) from beef heart mitochondria,^[^ ^34^ ^]^ oxidized ferredoxin (Fd_ox_) from *Pyrococcus furiosus* (Pc)^[^ ^33^ ^]^ and ispG from *Thermus thermophilus* ^[^ ^35^ ^]^ at the same excitation wavelength.

The most intense band at 335 cm^−1^ is attributed to the totally symmetric bridging vibration (or cluster‐breathing mode). Other bridging modes probably contribute at 378, 267, and 248 cm^−1^. Only two terminal Fe–S modes are observed at 407 and 357 cm^−1^. The position of the peaks is similar to those reported for *Thermus thermophilus* IspG, another enzyme of the MEP pathway (see Table 1) that also exhibits a [4Fe‐4S]^2+^ cluster of C3v symmetry, with an apical Fe atom bound to a glutamate residue.^[^ ^35^ ^,^ ^36^ ^]^ Interestingly, the totally symmetric bridging vibration frequency values, i.e., 335 and 336 cm^−1^ for IspH and IspG, respectively, unexpectedly fall in the range usually observed for clusters with complete cysteinyl coordination.^[^ ^33^ ^]^ IspH and IspG are thus two exceptions to the rule that clusters with an anomalous coordination of a specific iron atom are characterized by higher frequencies for the totally symmetric bridging vibration. The totally symmetric bridging vibration frequency is indeed observed at 339 cm^−1^ for the [4Fe‐4S]^2+^ cluster in aconitase from beef heart mitochondria, where the apical iron atom is coordinated to one hydroxyl group,^[^ ^34^ ^]^ and 342 cm^−1^ for the ferredoxin from *Pyrococcus furiosus*, where the fourth Fe atom is coordinated to an aspartate residue.^[^ ^33^ ^]^

### 2.2 Isotopic Labeling Experiments

As mentioned before, no X‐ray structure of *E. coli* IspH harboring the unique iron site has been reported in the absence of the substrate. The apical Fe atom of *E. coli* IspH was proposed to be linked to three water molecules in its substrate‐free form on the basis of NRVS studies. Here, we have performed isotopic labeling to provide strong evidence that water molecules are ligands of the unique iron site. Although direct Fe–O vibrational modes are not observed in the Raman spectrum of *E. coli* IspH, replacing ^1^H_2_^16^O by ^2^H_2_^16^O or ^2^H_2_^18^O may lead to small shifts of the RR bands of IspH due to vibrational mixing with Fe–S modes. The cluster also likely interacts with hydrogen bond donors in its environment and the ^1^H/^2^H substitution perturbs the X—H…S hydrogen bonds and thus indirectly the Fe—S stretching frequencies.^[^ ^34^ ^,^ ^37^ ^,^ ^38^ ^]^

In ^2^H_2_O, most of the vibrational modes remain unchanged within the detection limit of the Raman spectrometer. Only the bridging modes at 248 and 378 cm^−1^ are downshifted by 1 cm^−1^ (see Figure 2B and Table 1). Small shifts in both directions were also reported before for ferredoxins^[^ ^37^ ^]^ and explained by hydrogen bond effects that can be stronger with either H or D atoms depending on the S atoms that are involved in the vibration. In H_2_^18^O (Figure 2C), more vibrational modes are affected than in ^2^H_2_O, namely the bridging modes at 248 and 378 cm^−1^ and the terminal mode at 357 cm^−1^ that are downshifted by 1 cm^−1^ and the terminal mode at 407 cm^−1^ that is upshifted by 1 cm^−1^. More importantly, the totally symmetric bridging vibration at 335 cm^−1^ is downshifted by 1 cm^−1^, which confirms that the cluster is interacting with H_2_O through direct coordination of the oxygen atom on the apical Fe atom.

### 2.3 Binding of the Enzyme Substrate

**Figure 3:**
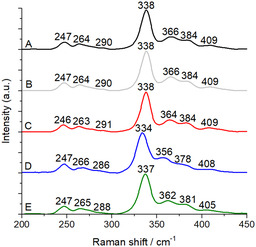
A) RR spectra of ≈0.3 mM IspH in the presence of four equivalents of the substrate HMBPP**,** B) HMBPP in H_2_^18^O, C) the inhibitor PropPP, D) the product IPP, and E) six equivalents of the product DMAPP measured with 457 nm laser excitation and 6 mW laser power at room temperature.

**Figure 4:**
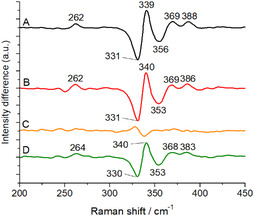
A) Difference RR spectra of IspH + HMBPP − IspH, B) IspH + PropPP − IspH, C) ispH + IPP − IspH, and D) IspH + DMAPP − IspH.

Upon HMBPP binding to IspH, all Fe–S bands in RR are shifting (see **Figure** **3A** and **Table** **2**). A downshift of 3 cm^−1^ is observed for the band at 267 cm^−1^ that was assigned to a bridging mode, and upshifts affect the terminal modes at 407 (+2) and 357 cm^−1^ (+9) and the bridging modes at 378 (+6) and 335 cm^−1^ (+3). The difference spectrum of IspH + HMBPP—IspH (see **Figure** **4A**) confirms that most of the Fe—S bands are altered when HMBPP binds to the cluster. This is fully consistent with a change in geometry of the apical Fe atom from an octahedral to a tetrahedral coordination that was revealed previously from Mössbauer spectroscopy^[^ ^14^ ^,^ ^15^ ^]^ and NRVS experiments.^[^ ^16^ ^]^ It is also consistent with the mode of binding of HMBPP that was established by X‐ray structure analysis.^[^ ^19^ ^]^ HMBPP interacts with the apical Fe atom through its hydroxyl group and is also involved in a 17‐member cyclic hydrogen bonding network between both its diphosphate and hydroxyl groups, Gln166, Thr168, Glu126, Thr167, and a water molecule. HMBPP may thus exert a ‘push’ or ‘pull’ effect on the apical iron, leading to changes in the Fe—S bond distances. Interestingly, the spectrum of *E. coli* IspH in the presence of HMBPP is not sensitive to the replacement of H_2_O by isotopically‐labeled H_2_^18^O (see Figure 3B), highlighting that also in solution all the water molecules coordinated to the apical Fe atom are removed when HMBPP is binding.

**Table 2:** Comparison of the Raman shifts (cm^−1^) observed for substrate‐free IspH and IspH in the presence of the substrate HMBPP, the inhibitor PropPP, and the two products isopentenyl diphosphate (IPP) and dimethylallyl diphosphate (DMAPP).

IspH	IspH + HMBPP	IspH + HMBPP/H_2_^18^O	IspH + PropPP	IspH + IPP	IspH + DMAPP
Mainly terminal Fe—S modes					
407	409	409	409	408	405
357	366	366	364	356	362
Mainly bridging Fe—S modes					
378	384	384	384	378	381
335	338	338	338	334	337
286	290	290	291	286	288
267	264	264	263	266	265
248	247	247	246	247	247

### 2.4 Binding of an Alkyne Diphosphate Inhibitor

Some alkyne diphosphates were shown to be potent competitive inhibitors of IspH, with propargyl diphosphate (PropPP) displaying an IC_50_ value of 6.7 µM for *A. aeolicus* IspH.^[^ ^39^ ^]^ According to several biophysical studies using Mössbauer spectroscopy, X‐ray crystallography^[^ ^40^ ^]^ and NRVS measurements^[^ ^41^ ^]^ on *E. coli* IspH, it was suggested that the apical Fe atom changes its ligand geometry from an octahedral to a tetrahedral coordination upon PropPP addition to the enzyme without binding of the alkyne group. It is believed that a single water molecule instead binds to the apical Fe atom, whereas the alkyne group remains distant from the cluster (See Scheme S1, Supporting Information).^[^ ^41^ ^]^ The coordination of the alkyne group to the apical Fe, with subsequent formation of a *π*/*σ* metallacycle, was, however, proposed for IspH from *A. aeolicus* in the reduced state on the basis of electron paramagnetic resonance and electron nuclear double resonance experiments.^[^ ^39^ ^]^

The RR spectrum of *E. coli* IspH in the presence of PropPP (Figure 3C) shows bands at positions comparable to the spectrum of IspH in complex with HMBPP (Figure 3A), and the difference between the spectra of IspH + PropPP and ligand‐free IspH (Figure 4B) is almost identical to the difference between the spectra of IspH + HMBPP and ligand‐free IspH (Figure 4A). This is a clear confirmation that the cluster adopts the same geometry in the presence of either PropPP or HMBPP, that the alkyne group does not directly bind to the apical Fe, even in solution, and favors a Fe–O coordination with most probably water as a direct ligand.

### 2.5 Binding of the Enzyme Products

To further analyze the versatile ligation state of the apical Fe atom of the cluster, we also compared the RR spectra of IspH in the presence of the products IPP or DMAPP (Figure 3D,E, respectively). X‐ray analysis showed that the double bond in neither of the two products could stabilize the apical Fe atom.^[^ ^19^ ^]^ In fact, a [3Fe‐4S] cluster was found in the resolved structures of *E. coli* IspH in complex with each of the two products. The Raman spectrum of *E. coli* IspH in the presence of IPP shows a very similar pattern to ligand‐free IspH, and as a consequence, the difference spectrum of IspH + IPP minus ligand‐free IspH is almost featureless (Figure 4C). The octahedral coordination geometry of the apical Fe atom is thus not altered when IPP is added, and an *η* ^2^‐binding mode with the C=C double bond of IPP is very unlikely.

IspH in the presence of DMAPP shows a different behavior (Figure 3E). The bands are located at intermediate positions between those of ligand‐free IspH and IspH in complex with HMBPP. This is not the spectrum expected for a [3Fe‐4S] cluster that exhibits only three bridging Fe–S modes and the totally symmetric vibration at higher wavenumbers.^[^ ^33^ ^,^ ^34^ ^,^ ^42^ ^]^ The apical iron is thus still present, but it has probably lost its octahedral coordination geometry. The difference between the spectra of IspH in the presence of DMAPP and ligand‐free IspH (Figure 4D) confirms that, unlike IPP, most Fe–S modes are altered when DMAPP is added.

## 3 Conclusion

In this study, we have characterized the unusual [4Fe‐4S]^2+^ cluster of *E. coli* IspH by RR spectroscopy that allowed us to probe the changes in geometry of the apical Fe atom upon binding of the enzyme substrate, an alkyne diphosphate inhibitor, and the two enzyme products, respectively. The cluster shows a series of bands in the 200–450 cm^−1^ range that can all be attributed to Fe–S modes. The bands are observed at positions very similar to those reported previously for IspG from *T. thermophilus* that also exhibits a [4Fe‐4S]^2+^ cluster of C3v symmetry. This latter enzyme belongs also to the MEP pathway and catalyzes a similar reaction than the IspH. Interestingly, the RR spectra of both enzymes display different features from those reported for [4Fe‐4S]^2+^ cluster harboring a non‐cysteinyl coordinated Fe site. More importantly, isotopic labeling experiments with H_2_^18^O bring the final evidence that the apical Fe atom is linked to H_2_O molecules, confirming the results of previous NRVS investigations. When the HMBPP substrate or the PropPP inhibitor is added, a dramatic change of the spectrum occurs that is consistent with a change of coordination geometry of the apical Fe atom from octahedral to tetrahedral and a Fe–O coordination that was suggested before on the basis of Mössbauer spectroscopy and NRVS experiments. The loss of octahedral coordination of the apical Fe atom is also very likely when the DMAPP product is added, while no change is observed with IPP. These distinct binding modes may play a key role in the catalytic outcome of the enzyme by modulating product distribution. Specifically, the preferential stabilization or release of one product over the other could account for the IPP:DMAPP ratio of about 6:1 observed during catalysis.

## 4 Experimental Section

4.1

4.1.1

##### Materials

All chemicals were purchased from Sigma–Aldrich, Co., and were of high purity grade. High‐density polyethylene plates (PEs) were obtained from Reichelt Chemietechnik Co. Air‐tight plastic (low‐density polyethylene) bags were purchased from Fisher Scientific Co.

##### Product Synthesis

HMBPP was synthesized according to the route we have described before.^[^ ^43^ ^]^ IPP and DMAPP were obtained starting from isopentenol and dimethylallyl alcohol, respectively, using the phosphorylation method described by Keller and Thompson.^[^ ^44^ ^]^ PropPP was synthesized as described previously.^[^ ^39^ ^]^

##### Protein Production and Purification

IspH was produced from *E. coli* M15‐[pREP4, pQE30‐IspH] strains and purified by affinity chromatography as described before.^[^ ^45^ ^]^ The IspH concentration was determined by the method of Bradford^[^ ^46^ ^]^ with bovine serum albumin as a standard. The iron content per enzyme was determined by the method of Fish,^[^ ^47^ ^]^ and the sulfur content per enzyme by the method of Beinert.^[^ ^48^ ^]^ Iron and sulfur contents were similar to the values reported previously.^[^ ^15^ ^]^ The IspH activity was measured in the conditions described previously^[^ ^49^ ^]^ and it was found to be around 1000 nmol min^−1 ^mg^−1^ in agreement with previous reports.^[^ ^45^ ^,^ ^49^ ^]^ The enzyme was prepared at a concentration of 14 mg mL^−1^ (0.39 mM) in 50 mM Tris‐HCl, pH 8, and stored in aliquots of 40–500 μL in liquid nitrogen until usage.

##### Raman Spectroscopy

For the measurement of the RR spectra, enzyme solutions with a concentration between 11 and 16 mg mL^−1^ (or 0.3–0.4 mM) were used. A dry film was prepared in the glove box by depositing 3 µL of the enzyme solution on a high‐density PE. The solution was dried between 20 and 30 min in the glove box, and under reduced pressure if necessary. The PE plate was introduced in an air‐tight plastic bag before being taken out of the glove box, transported, and measured. In this way, all measurements could be conducted under anaerobic conditions. All spectra were measured on an inVia Renishaw spectrometer coupled with a thermoelectrically‐cooled charge‐coupled device as a detector. An argon excitation laser with a wavelength of 457 nm and a power of 5–6 mW was used. For focusing, a 50X/0.75 NA dipping microscope objective (Leica) was used. The spectrometer was calibrated with a silicon plate before each usage. 30–50 scans with an exposure time of 10 s were measured in the 200–550 cm^−1^ range at room temperature. The spectra were treated with the software Origin 9.0. The baseline was corrected manually. The averaged spectra were further smoothed with the fast fourier transform filter method from the software Origin using eight points and the peak positions were determined with the help of the second derivative of the spectra. Difference RR spectra were calculated with the software Origin by overlaying two RR spectra and adjusting their intensities so that as few differences as possible were observed between the two spectra. The two spectra were then subtracted from each other. The best signal‐to‐noise ratio was obtained with dry films of proteins. In order to make sure that drying the protein did not lead to alteration of the spectra, we also measured the spectrum in solution using a capillary. The obtained spectrum (see Figure S1, Supporting Information) shows a lower signal‐to‐noise ratio, but the bands are observed at the same position.

##### Isotopic Exchange

Isotopic exchange with ^2^H_2_O or with H_2_^18^O was conducted by buffer exchange. 40 µL of an IspH solution was added to a 50 mM TrisHCl solution in ^2^H_2_O or in H_2_^18^O. Then, the respective solution was incubated for 24 h at 4 °C (dry bath). The solutions were concentrated using a centrifugal filter with a cutoff at 10 kDa (Amicon Ultra, 0.5 mL) until their concentration was above 10 mg mL^−1^ (0.28 mM). The dry films were prepared as before.

##### Addition of Ligands

The IspH solution was mixed with four equivalents of the substrate HMBPP, PropPP, or one of the two products IPP and DMAPP, respectively. For DMAPP, six equivalents were used. 3 µL of this solution was deposited on a high‐density PE plate to prepare the dry film as described before.

## Conflict of Interest

The authors declare no conflict of interest.

## Supporting information

Supplementary Material

## Data Availability

The data that support the findings of this study are available from the corresponding author upon reasonable request.

## References

[1] C. S. Ho , C. T. H. Wong , T. T. Aung , R. Lakshminarayanan , J. S. Mehta , S. Rauz , A. McNally , B. Kintses , S. J. Peacock , C. de la Fuente‐Nunez , R. E. W. Hancock , D. S. J. Ting , Lancet Microbe 2025, 6, 100947. 39305919 10.1016/j.lanmic.2024.07.010

[2] L. C. J. Murray , et al., Lancet 2022, 399, 629. 35065702 10.1016/S0140-6736(21)02724-0 PMC8841637

[3] J. O’Neill , Tackling Drug‐Resistant Infections Globally: Final Report and Recommendations 2016, https://amr‐review.org/sites/default/files/160518_Final%20paper_with%20cover.pdf (accessed: May 2025).

[4] E. D. Brown , G. D. Wright , Chem. Rev. 2005, 105, 759. 15700964 10.1021/cr030116o

[5] M. Rohmer , C. Grosdemange‐Billiard , M. Seemann , D. Tritsch , Curr. Opin. Investig. Drugs 2004, 5, 154. 15043389

[6] T. Masini , A. K. H. Hirsch , J. Med. Chem. 2014, 57, 9740. 25210872 10.1021/jm5010978

[7] A. Frank , M. Groll , Chem. Rev. 2017, 117, 5675. 27995802 10.1021/acs.chemrev.6b00537

[8] A. Allamand , T. Piechowiak , D. Lièvremont , M. Rohmer , C. Grosdemange‐Billiard , Molecules 2023, 28, 1403. 36771066 10.3390/molecules28031403 PMC9919496

[9] H. Jobelius , G. I. Bianchino , F. Borel , P. Chaignon , M. Seemann , Molecules 2022, 27, 708. 35163971 10.3390/molecules27030708 PMC8837944

[10] M. Wolff , M. Seemann , B. T. S. Bui , Y. Frapart , D. Tritsch , A. G. Estrabot , M. Rodríguez‐Concepción , A. Boronat , A. Marquet , M. Rohmer , FEBS Lett. 2003, 541, 115. 12706830 10.1016/s0014-5793(03)00317-x

[11] W. Xu , N. S. Lees , D. Hall , D. Welideniya , B. M. Hoffman , E. C. Duin , Biochemistry 2012, 51, 4835. 22646150 10.1021/bi3001215 PMC3426640

[12] H. Beinert , R. H. Holm , E. Münck , Science 1997, 277, 653. 9235882 10.1126/science.277.5326.653

[13] H. Beinert , J. Biol. Inorg. Chem. 2000, 5, 2. 10766431 10.1007/s007750050002

[14] Y. Xiao , L. Chu , Y. Sanakis , P. Liu , J. Am. Chem. Soc. 2009, 131, 9931. 19583210 10.1021/ja903778d

[15] M. Seemann , K. Janthawornpong , J. Schweizer , L. H. Böttger , A. Janoschka , A. Ahrens‐Botzong , E. N. Tambou , O. Rotthaus , A. X. Trautwein , M. Rohmer , V. Schünemann , J. Am. Chem. Soc. 2009, 131, 13184. 19708647 10.1021/ja9012408

[16] I. Faus , A. Reinhard , S. Rackwitz , J. A. Wolny , K. Schlage , H.‐C. Wille , A. Chumakov , S. Krasutsky , P. Chaignon , C. D. Poulter , M. Seemann , V. Schünemann , Angew. Chem., Int. Ed. 2015, 54, 12584. 10.1002/anie.201502494 PMC4609541 26118554

[17] I. Rekittke , J. Wiesner , R. Röhrich , U. Demmer , E. Warkentin , W. Xu , K. Troschke , M. Hintz , J. H. No , E. C. Duin , E. Oldfield , H. Jomaa , U. Ermler , J. Am. Chem. Soc. 2008, 130, 17206. 19035630 10.1021/ja806668q PMC2756146

[18] T. Gräwert , F. Rohdich , I. Span , A. Bacher , W. Eisenreich , J. Eppinger , M. Groll , Angew. Chem., Int. Ed. 2009, 48, 5756. 10.1002/anie.200900548 19569147

[19] T. Gräwert , I. Span , W. Eisenreich , F. Rohdich , J. Eppinger , A. Bacher , M. Groll , Proc. Natl. Acad. Sci. 2010, 107, 1077. 20080550 10.1073/pnas.0913045107 PMC2824267

[20] I. Span , K. Wang , W. Wang , J. Jauch , W. Eisenreich , A. Bacher , E. Oldfield , M. Groll , Angew. Chem., Int. Ed. 2013, 52, 2118. 10.1002/anie.201208469 PMC3734547 23307751

[21] I. Span , K. Wang , W. Eisenreich , A. Bacher , Y. Zhang , E. Oldfield , M. Groll , J. Am. Chem. Soc. 2014, 136, 7926. 24813236 10.1021/ja501127j PMC4063180

[22] W. Wang , K. Wang , I. Span , J. Jauch , A. Bacher , M. Groll , E. Oldfield , J. Am. Chem. Soc. 2012, 134, 11225. 22687151 10.1021/ja303445z PMC3394908

[23] S. Ghebreamlak , S. A. Stoian , N. S. Lees , B. Cronin , F. Smith , M. O. Ross , J. Telser , B. M. Hoffman , E. C. Duin , J. Am. Chem. Soc. 2024, 146, 3926. 38291562 10.1021/jacs.3c11674

[24] C. A. Citron , N. L. Brock , P. Rabe , J. S. Dickschat , Angew. Chem., Int. Ed. 2012, 51, 4053. 10.1002/anie.201201110 22411616

[25] I. Span , T. Gräwert , A. Bacher , W. Eisenreich , M. Groll , J. Mol. Biol. 2012, 416, 1. 22137895 10.1016/j.jmb.2011.11.033

[26] P. G. Blachly , G. M. Sandala , D. A. Giammona , D. Bashford , J. A. McCammon , L. Noodleman , Inorg. Chem. 2015, 54, 6439. 26098647 10.1021/acs.inorgchem.5b00751 PMC4568833

[27] P. Chaignon , B. E. Petit , B. Vincent , L. Allouche , M. Seemann , Chem. Eur. J. 2020, 26, 1032. 31756006 10.1002/chem.201904676

[28] F. Rohdich , F. Zepeck , P. Adam , S. Hecht , J. Kaiser , R. Laupitz , T. Gräwert , S. Amslinger , W. Eisenreich , A. Bacher , D. Arigoni , Proc. Natl. Acad. Sci. 2003, 100, 1586. 12571359 10.1073/pnas.0337742100 PMC149876

[29] R. S. Czernuszewicz , K. A. Macor , M. K. Johnson , A. Gewirth , T. G. Spiro , J. Am. Chem. Soc. 1987, 109, 7178.

[30] S. Todorovic , M. Teixeira , J. Biol. Inorg. Chem. 2018, 23, 647. 29368020 10.1007/s00775-018-1533-0 PMC6006211

[31] G. Caserta , L. Zuccarello , C. Barbosa , C. M. Silveira , E. Moe , S. Katz , P. Hildebrandt , I. Zebger , S. Todorovic , Coord. Chem. Rev. 2022, 452, 214287.

[32] W. Fu , S. O’Handley , R. P. Cunningham , M. K. Johnson , J. Biol. Chem. 1992, 267, 16135. 1644800

[33] R. C. Conover , A. T. Kowal , W. G. Fu , J. B. Park , S. Aono , M. W. Adams , M. K. Johnson , J. Biol. Chem. 1990, 265, 8533. 2160461

[34] L. K. Kilpatrick , M. C. Kennedy , H. Beinert , R. S. Czernuszewicz , T. G. Spiro , D. Qiu , J. Am. Chem. Soc. 1994, 116, 4053.

[35] D. Adedeji , H. Hernandez , J. Wiesner , U. Köhler , H. Jomaa , E. C. Duin , FEBS Lett. 2007, 581, 279. 17214985 10.1016/j.febslet.2006.12.026

[36] I. Rekittke , T. Nonaka , J. Wiesner , U. Demmer , E. Warkentin , H. Jomaa , U. Ermler , FEBS Lett. 2011, 585, 447. 21167158 10.1016/j.febslet.2010.12.012

[37] G. Backes , Y. Mino , T. M. Loehr , T. E. Meyer , M. A. Cusanovich , W. V. Sweeney , E. T. Adman , J. Sanders‐Loehr , J. Am. Chem. Soc. 1991, 113, 2055.

[38] Y. Mino , T. M. Loehr , K. Wada , H. Matsubara , J. Sanders‐Loehr , Biochemistry 1987, 26, 8059. 3442645 10.1021/bi00399a006

[39] K. Wang , W. Wang , J.‐H. No , Y. Zhang , Y. Zhang , E. Oldfield , J. Am. Chem. Soc. 2010, 132, 6719. 20426416 10.1021/ja909664j PMC2874468

[40] I. Span , K. Wang , W. Wang , Y. Zhang , A. Bacher , W. Eisenreich , K. Li , C. Schulz , E. Oldfield , M. Groll , Nat. Commun. 2012, 3, 1042. 22948824 10.1038/ncomms2052 PMC3745992

[41] B. O’Dowd , S. Williams , H. Wang , J. H. No , G. Rao , W. Wang , J. A. McCammon , S. P. Cramer , E. Oldfield , ChemBioChem 2017, 18, 914. 28253432 10.1002/cbic.201700052 PMC5445010

[42] M. K. Johnson , R. S. Czernuszewicz , T. G. Spiro , J. A. Fee , W. V. Sweeney , J. Am. Chem. Soc. 1983, 105, 6671.

[43] M. Wolff , M. Seemann , C. Grosdemange‐Billiard , D. Tritsch , N. Campos , M. Rodríguez‐Concepción , A. Boronat , M. Rohmer , Tetrahedron Lett. 2002, 43, 2555.

[44] R. K. Keller , R. Thompson , J. Chromatogr. A. 1993, 645, 161. 10.1016/0021-9673(93)80630-q 23738391

[45] K. Janthawornpong , S. Krasutsky , P. Chaignon , M. Rohmer , C. D. Poulter , M. Seemann , J. Am. Chem. Soc. 2013, 135, 1816. 23316732 10.1021/ja309557s PMC3644560

[46] M. M. Bradford , Anal. Biochem. 1976, 72, 248. 942051 10.1016/0003-2697(76)90527-3

[47] W. W. Fish , Methods Enzymol. 1988, 158, 357. 3374387 10.1016/0076-6879(88)58067-9

[48] H. Beinert , Anal. Biochem. 1983, 131, 373. 6614472 10.1016/0003-2697(83)90186-0

[49] B. E. Petit , H. Jobelius , G. I. Bianchino , M. Guérin , F. Borel , P. Chaignon , M. Seemann , C. R. Chim. 2023, 26, 79.

