# Supplementary material for: Resonance Raman Spectroscopic Study of the Unusual [4Fe‐4S]2+ Cluster of IspH, the Last Enzyme of the Methylerythritol Phosphate Pathway for Terpenoid Biosynthesis
Source: Chembiochem. 2025 Aug 26;26(18):e202500428. doi: 10.1002/cbic.202500428 (PMC12447371; doi:10.1002/cbic.202500428)
Supplement: Supplementary file 1 — Supplementary Material [file CBIC-26-e202500428-s001.pdf]

Supporting Information for

Resonance Raman Spectroscopic Study of the Unusual $[4Fe\text{-}4S]^{2+}$ Cluster of IspH, the Last Enzyme of the Methylerythritol Phosphate Pathway for Terpenoid Biosynthesis

Hannah Jobelius,[a] Philippe Chaignon,[a] Gabriella I. Bianchino,[a] Joanna Wandzig,[a] Petra Hellwig,[b,c] Myriam Seemann,* [a] and Frederic Melin*[b]

[a] Dr. H. Jobelius, Dr. P. Chaignon, Dr. G. I. Bianchino, Dr. J. Wandzig, Dr. M. Seemann
Equipe Chimie Biologique et Applications Thérapeutiques
Institut de Chimie de Strasbourg, UMR 7177 CNRS
67000 Strasbourg, France
E-mail: mseemann@unistra.fr

[b] Prof. Dr. P. Hellwig, Dr. F. Melin
Laboratoire de Bioélectrochimie et Spectroscopie, UMR 7140, Chimie de la Matière Complexe
Université de Strasbourg CNRS
67000 Strasbourg, France
E-mail: fmelin@unistra.fr

[c] Prof. Dr. P. Hellwig
Institut Universitaire de France (IUF)

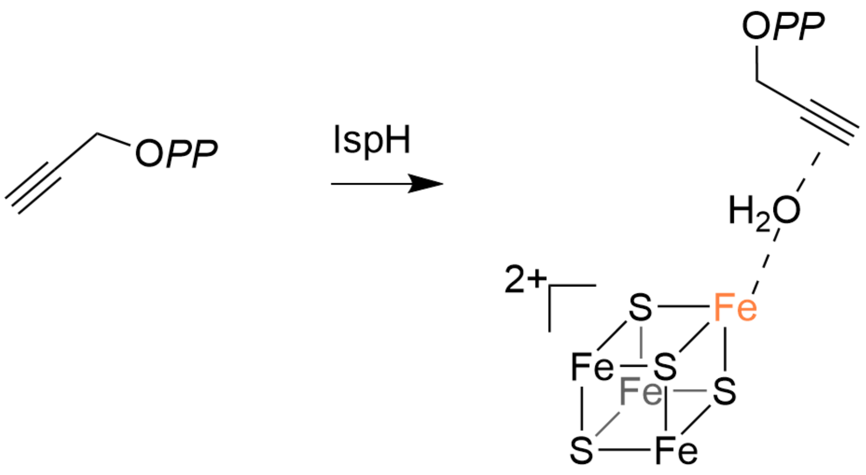

Scheme S1. Proposed mode of interaction between IspH in the oxidized state and the inhibitor PropPP.

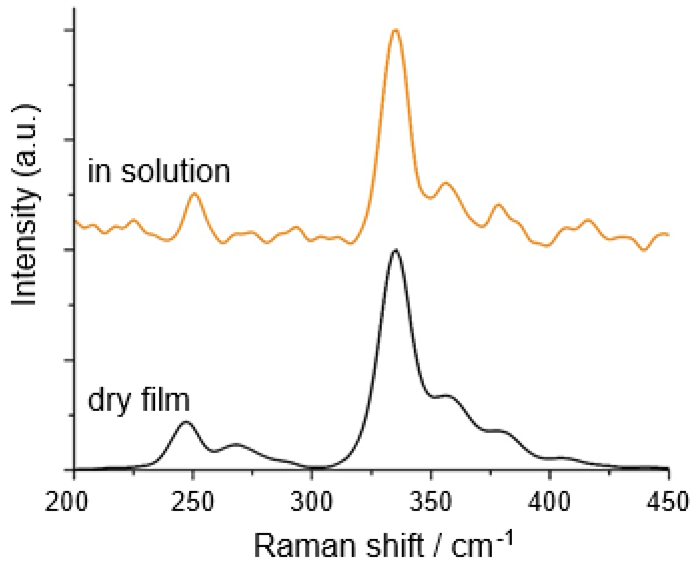

Figure S1. Comparison between the resonance Raman spectra of approximately 0.3 mM IspH measured in solution or as a dry film with 457 nm laser excitation and 6 mW laser power at room temperature
